# Supplementary material for: Sustainable 3D printing by reversible salting-out effects with aqueous salt solutions
Source: Nat Commun. 2024 May 9;15:3925. doi: 10.1038/s41467-024-48121-7 (PMC11082145; doi:10.1038/s41467-024-48121-7)
Supplement: Supplementary file 1 — Supplementary Information [file 41467_2024_48121_MOESM1_ESM.pdf]

Supplementary Information

Sustainable 3D printing by reversible salting-out effects with aqueous salt solutions

Donghwan Ji[1], Joseph Liu[1], Jiayu Zhao[1], Minghao Li[2], Yumi Rho[1,3], Hwansoo Shin[4], Tae Hee Han[4], Jinhye Bae[1,2,3]*

[1]Department of NanoEngineering, University of California San Diego, La Jolla, CA, 92093, United States

[2]Materials Science and Engineering Program, University of California San Diego, La Jolla, CA, 92093, United States

[3]Chemical Engineering Program, University of California San Diego, La Jolla, CA, 92093, United States

[4]Department of Organic and Nano Engineering and Human-Tech Convergence Program, Hanyang University, Seoul 04763, Republic of Korea

*Correspondence to: Prof. Jinhye Bae (E-mail: j3bae@ucsd.edu)

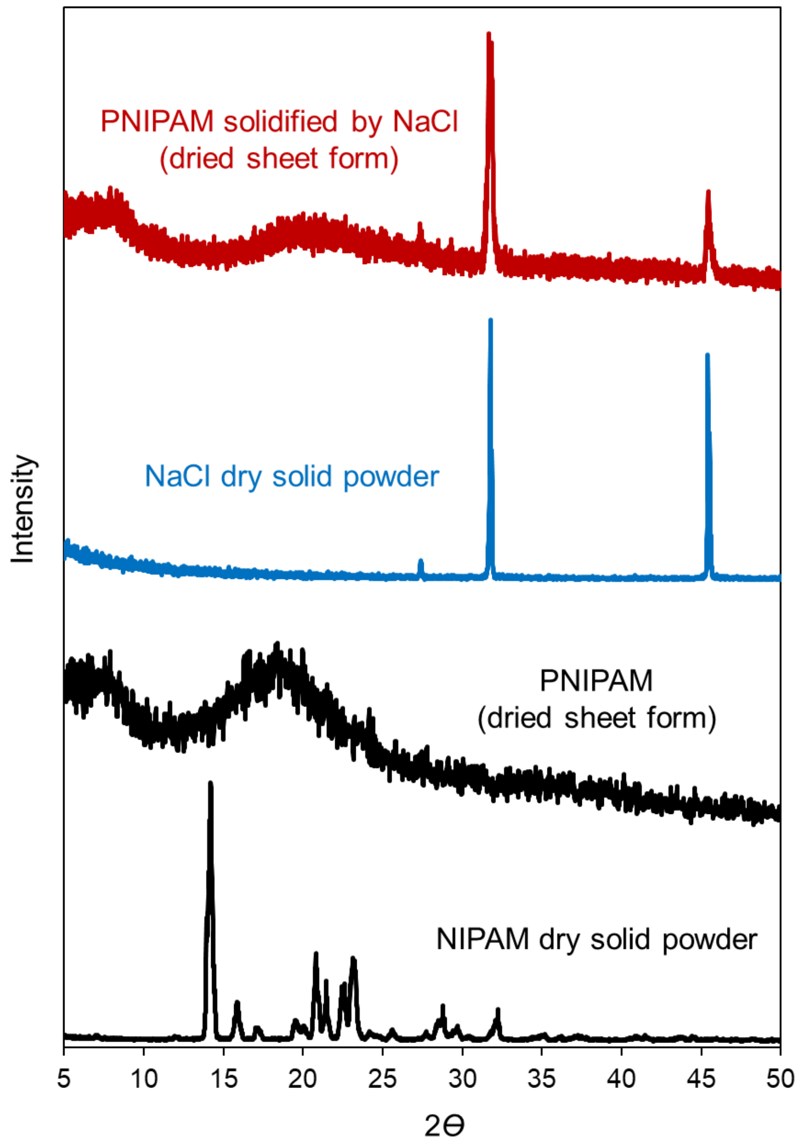

Supplementary Fig. 1. XRD pattern of NIPAM, PNIPAM, NaCl, and solidified PNIPAM by NaCl, respectively. Once NIPAM dry solid powder (as received from the manufacturer) dissolved and polymerized, the PNIPAM was in an amorphous state. The PNIPAM solidified by NaCl was also amorphous, whose XRD pattern was similar to that of the PNIPAM. The sharp peaks observed in the solidified PNIPAM sample were from NaCl crystals.

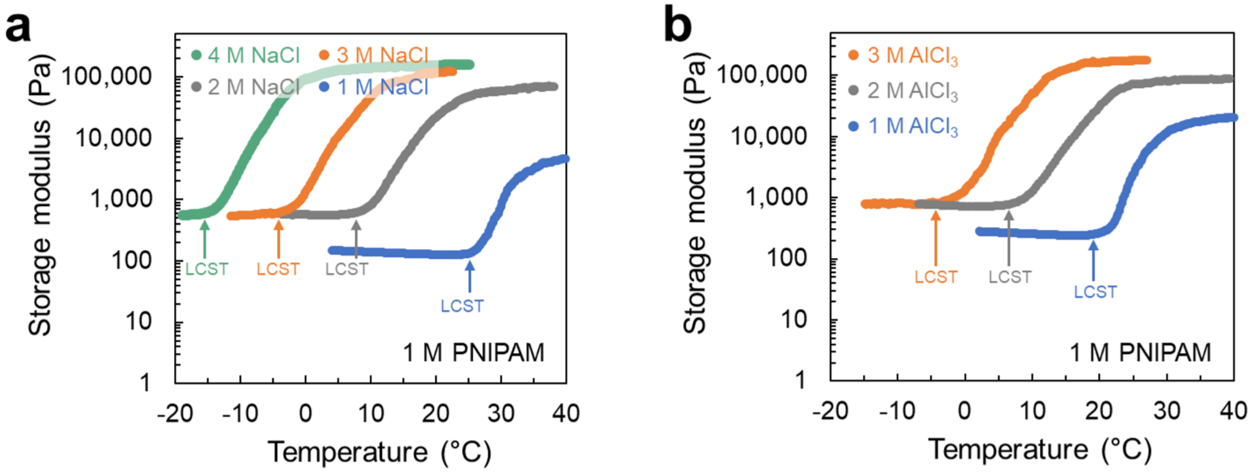

Supplementary Fig. 2. Shift in phase transition temperature, LCST, at different salt ions and their concentrations. (a) Storage moduli change of 1 M PNIPAM under different NaCl concentrations, over the temperature increase. (b) Storage moduli change of 1 M PNIPAM under different $AlCl_3$ concentrations, over the temperature increase. The PNIPAM solidified within a 4 M $AlCl_3$ solution was not fully dissolved on the rheometer stage of −20 °C which was the minimum setting-temperature of the rheometer.

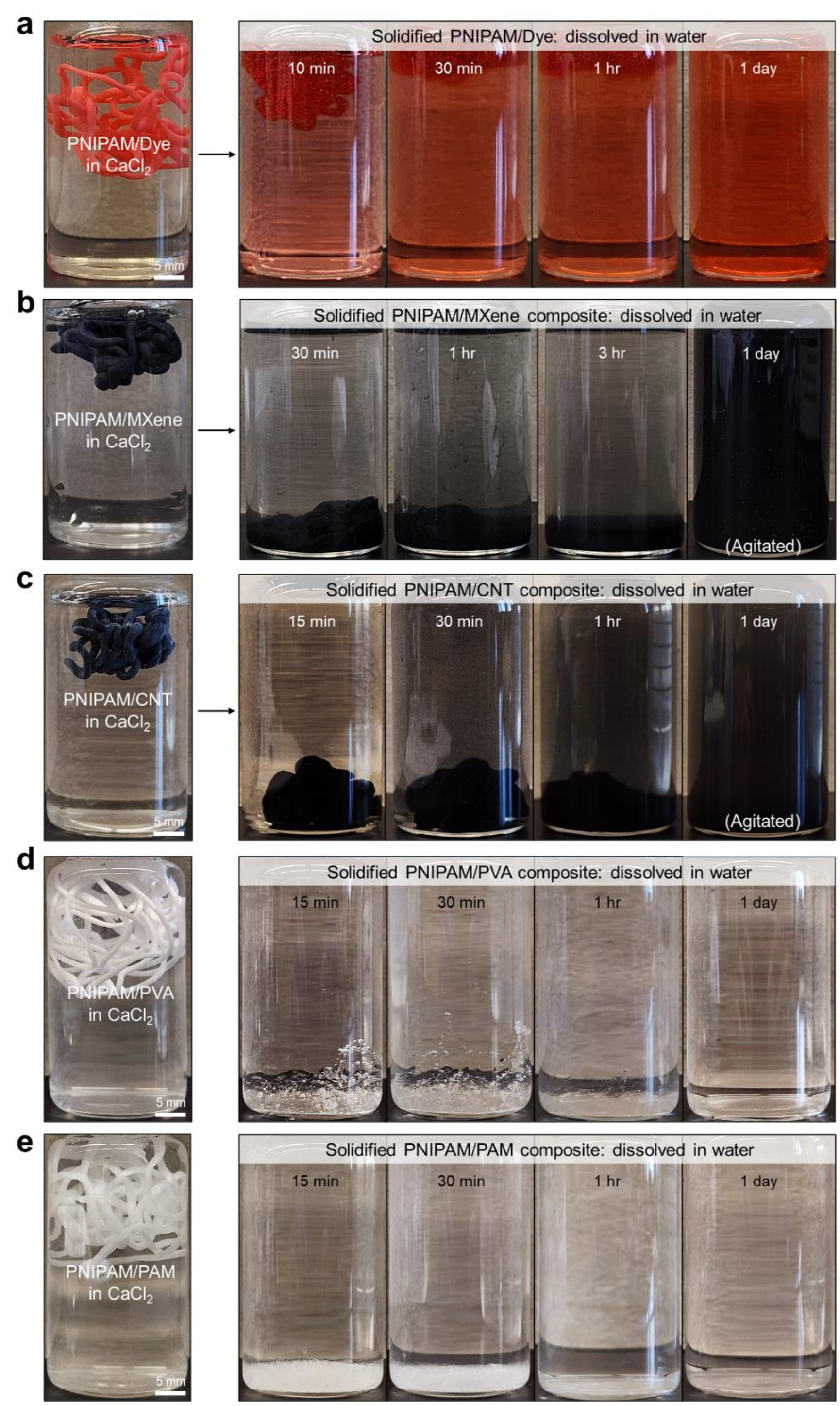

Supplementary Fig. 3. Water-dissolution processes of solidified PNIPAM-based composite over time. (a) PNIPAM/Dye, (b) PNIPAM/MXene, (c) PNIPAM/CNT, (d) PNIPAM/PVA, and (e) PNIPAM/PAM composite systems, which solidified in 3 M $CaCl_2$ solution, all became fully dissolved in water, similar to pure PNIPAM.

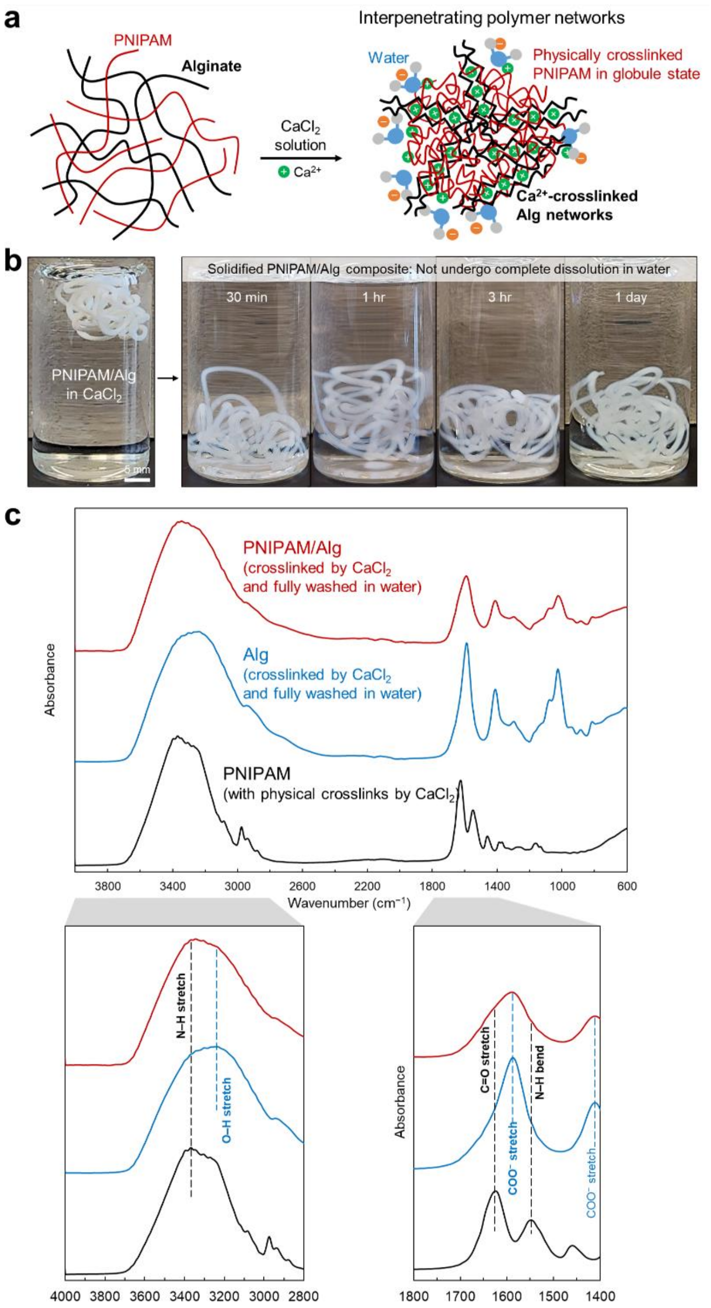

Supplementary Fig. 4. PNIPAM/Alg composite forming interpenetrating polymer networks. (a) Schematic depicting the formation of interpenetrating polymer networks comprising aggregated and physically crosslinked PNIPAM chains and Ca^{2+}-crosslinked Alg networks. (b) Solidified PNIPAM/Alg in 3 M $CaCl_2$ solution and its stability in water. (c) FTIR patterns of PNIPAM crosslinked by $CaCl_2$, Alg crosslinked by $CaCl_2$ and thoroughly washed in water, and PNIPAM/Alg crosslinked by $CaCl_2$ and thoroughly washed in water. These FTIR patterns were collected by measuring samples dried at 25 °C.

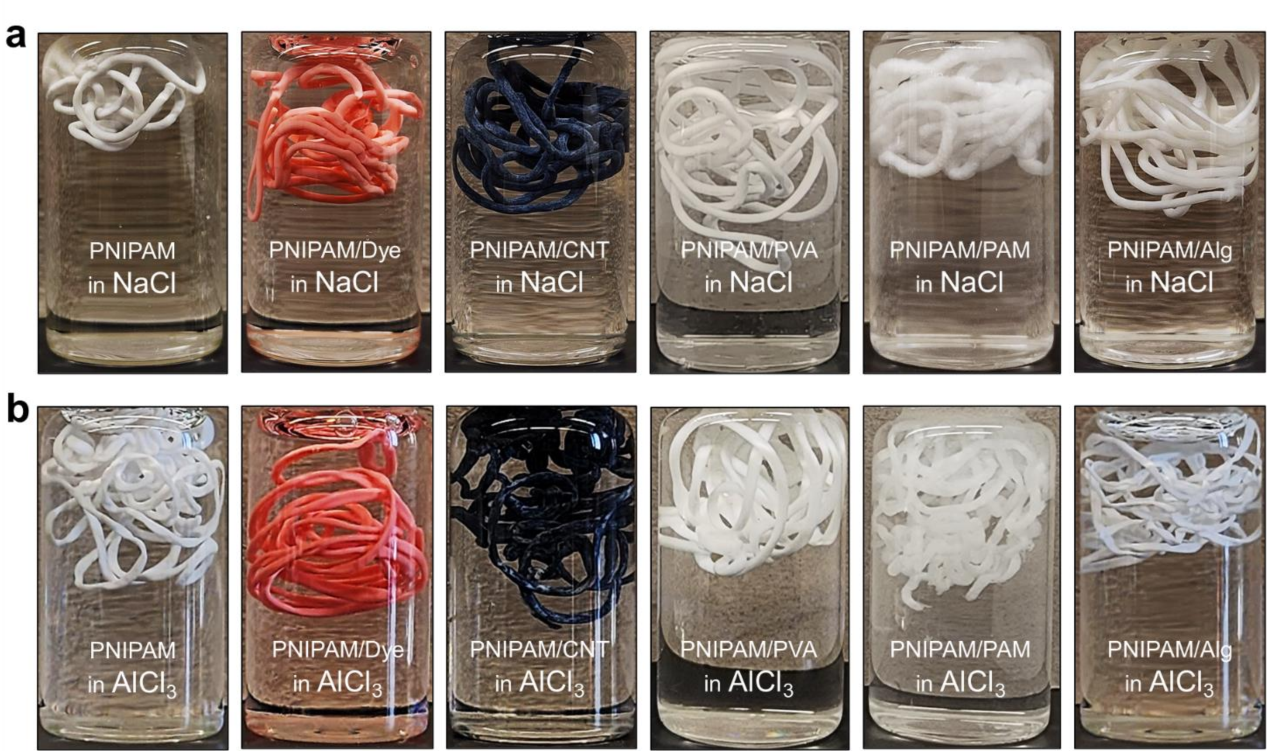

Supplementary Fig. 5. Solidification of PNIPAM-based composite solutions in other aqueous salt solutions. (a) 3 M NaCl solution. (b) 3 M $AlCl_3$ solution.

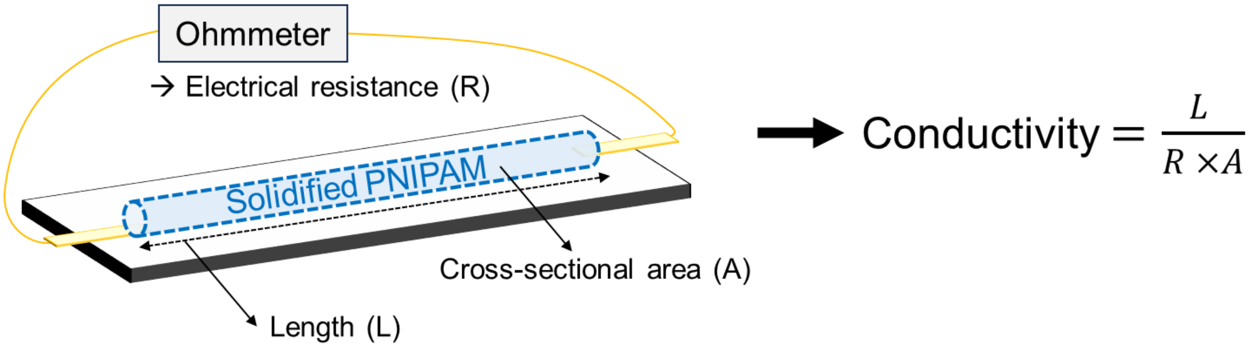

Supplementary Fig. 6. Measurement setup for the conductivity of solidified PNIPAM and PNIPAM/MXene+CNT. To demonstrate the effect of conductive inorganic particles (MXene and CNT) on the conductivity enhancement, we compared pure PNIPAM and PNIPAM/MXene+CNT composite representatively. Each solution was extruded into the 3 M $CaCl_2$ solution, resulting in fully crosslinked and solidified samples. Water on the surface of the solidified samples was gently wiped using Kimtech Wipes, and the sample was placed on the customized measuring device as shown in the schematic illustration.

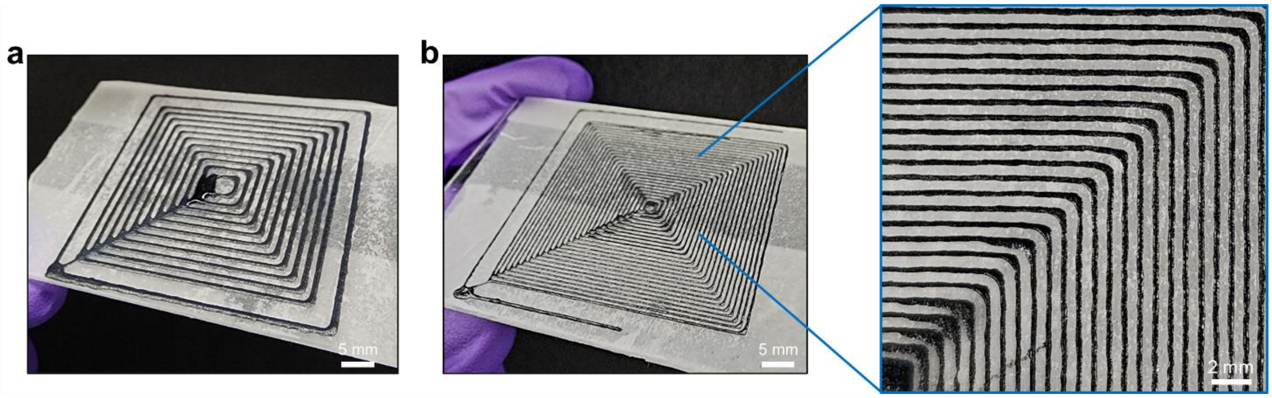

Supplementary Fig. 7. Finely printed structures of PNIPAM/CNT composite using different nozzle sizes. The structure was printed using (a) a 0.6 mm-diameter nozzle and (b) a 0.25 mm-diameter nozzle, respectively. This fine and repetitive structure was consistently printed on a cm-scale (4 × 4 cm^2).

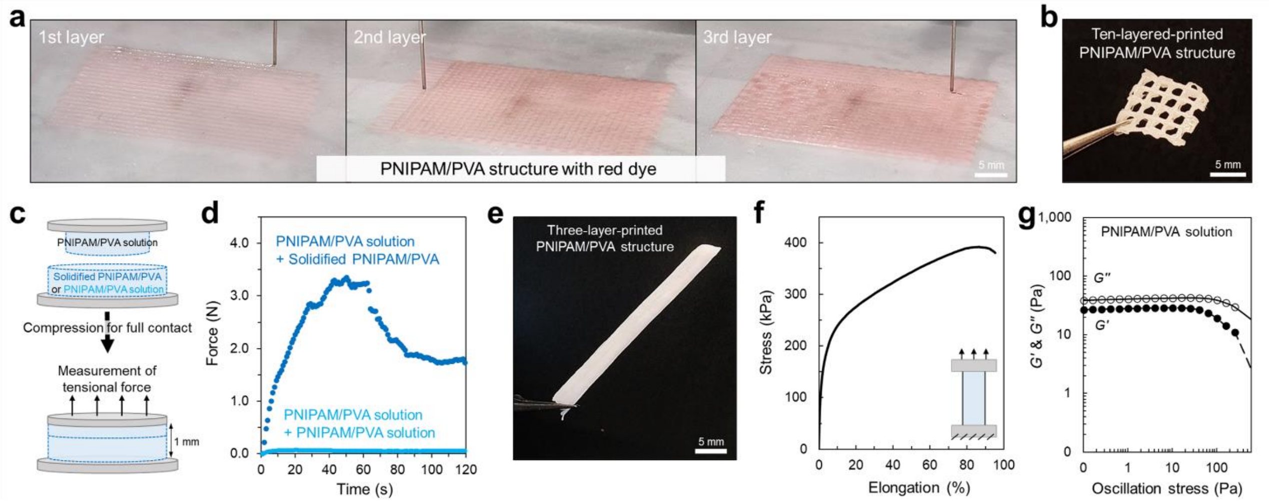

Supplementary Fig. 8. Printing of PNIPAM/PVA composite solution. (a) Photographs showing multi-layer printing of PNIPAM/PVA solution ink with red dye using a 200-µm-diameter nozzle. (b) Photograph of ten-layer printed grid structure of PNIPAM/PVA. (c) Schematic illustration depicting how we evaluated the adhesion force between the first and the second layers, and (d) the corresponding result. In the case of PNIPAM/PVA solution and solidified PNIPAM/PVA as the dark blue graph, diffusion of salt ions from the first layer (solidified PNIPAM/PVA) to the second layer (freshly extruded PNIPAM/PVA solution) likely formed physical crosslinks and entanglement of the polymers across both layers, thereby generating the tensional force, unlike the case of PNIPAM/PVA solutions at both (sky blue graph). The detailed measurement procedure is described in the Experimental section (Adhesion force measurement) of the main article. (e) Photograph of three-layer printed structure of PNIPAM/PVA and (f) its tensile stress-strain curve showing good physical and mechanical stability with free-standing and stretchable characteristics. (g) G' and G'' as a function of oscillation shear stress, demonstrating shear-yielding property of PNIPAM/PVA solution.

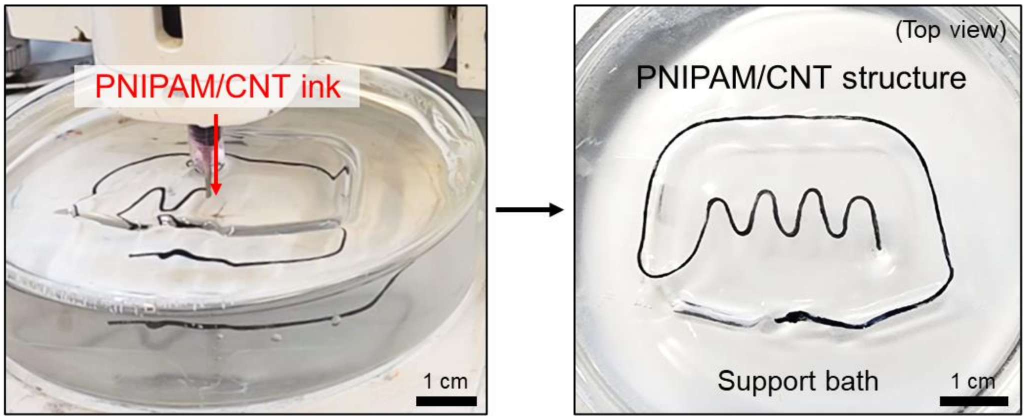

Supplementary Fig. 9. PNIPAM/CNT structure in the support bath. The printed solution immediately solidified upon contact with the salt ions dissolved in the support bath.

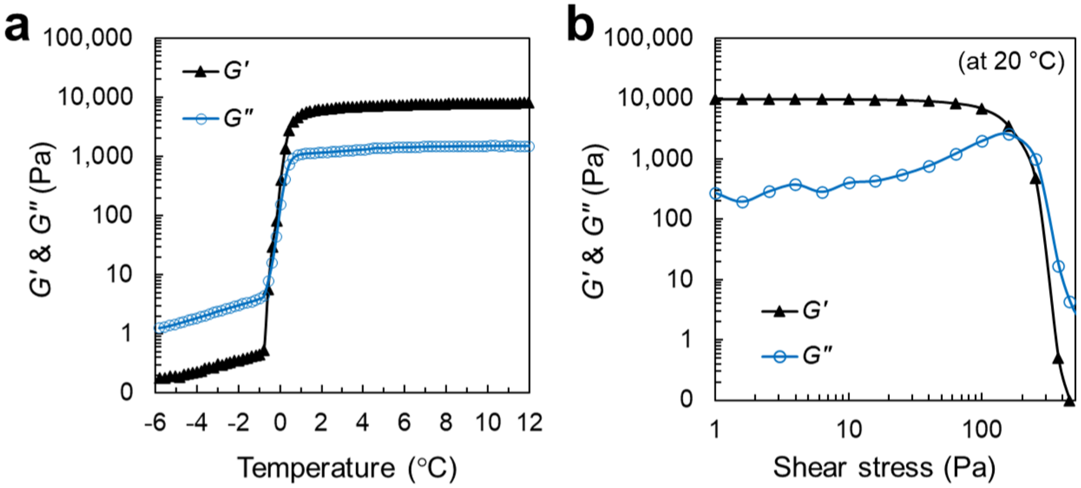

Supplementary Fig. 10. Rheological characteristics of Pluronic F-127 support bath containing 3 M $CaCl_2$. G' and G" (a) as a function of temperature and (b) as a function of shear stress. The liquid-state mixture was prepared at low temperatures and was a stable solid at ambient temperatures. The solid support bath temporarily became in a fluid-like state while subjected to shear stress (over yield point) generated by the syringe nozzle moving inside the bath.

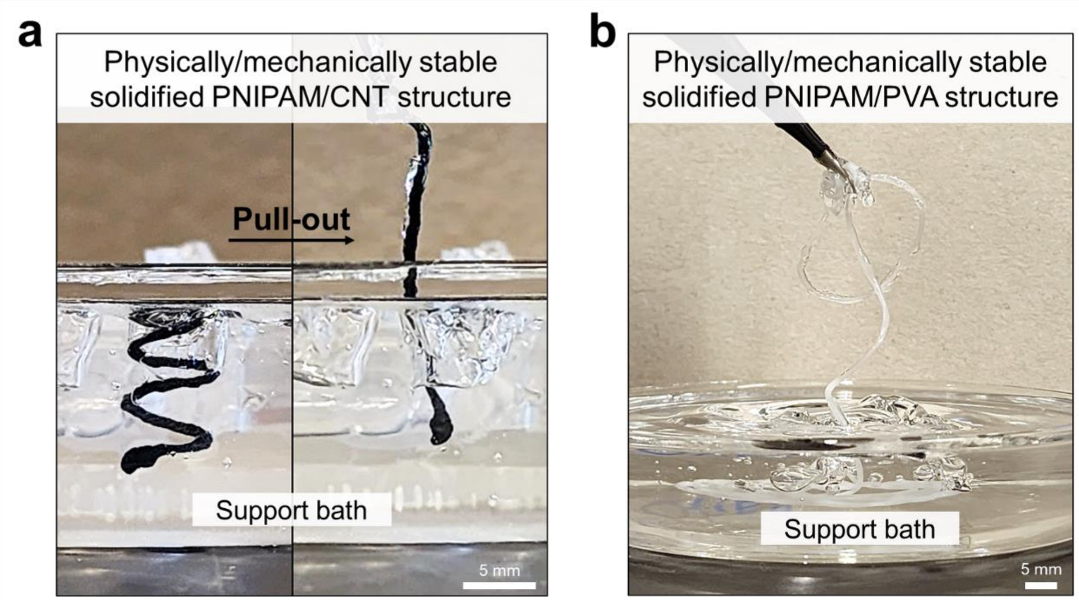

Supplementary Fig. 11. Physically/mechanically stable structures solidified in the support bath. (a) PNIPAM/CNT and (b) PNIPAM/PVA solutions were stably solidified in the bath during vertical and horizontal nozzle movements. The solidified sample was able to be pulled out from the support bath. The fast movement of the printing nozzle in the bath often made some air bubbles.

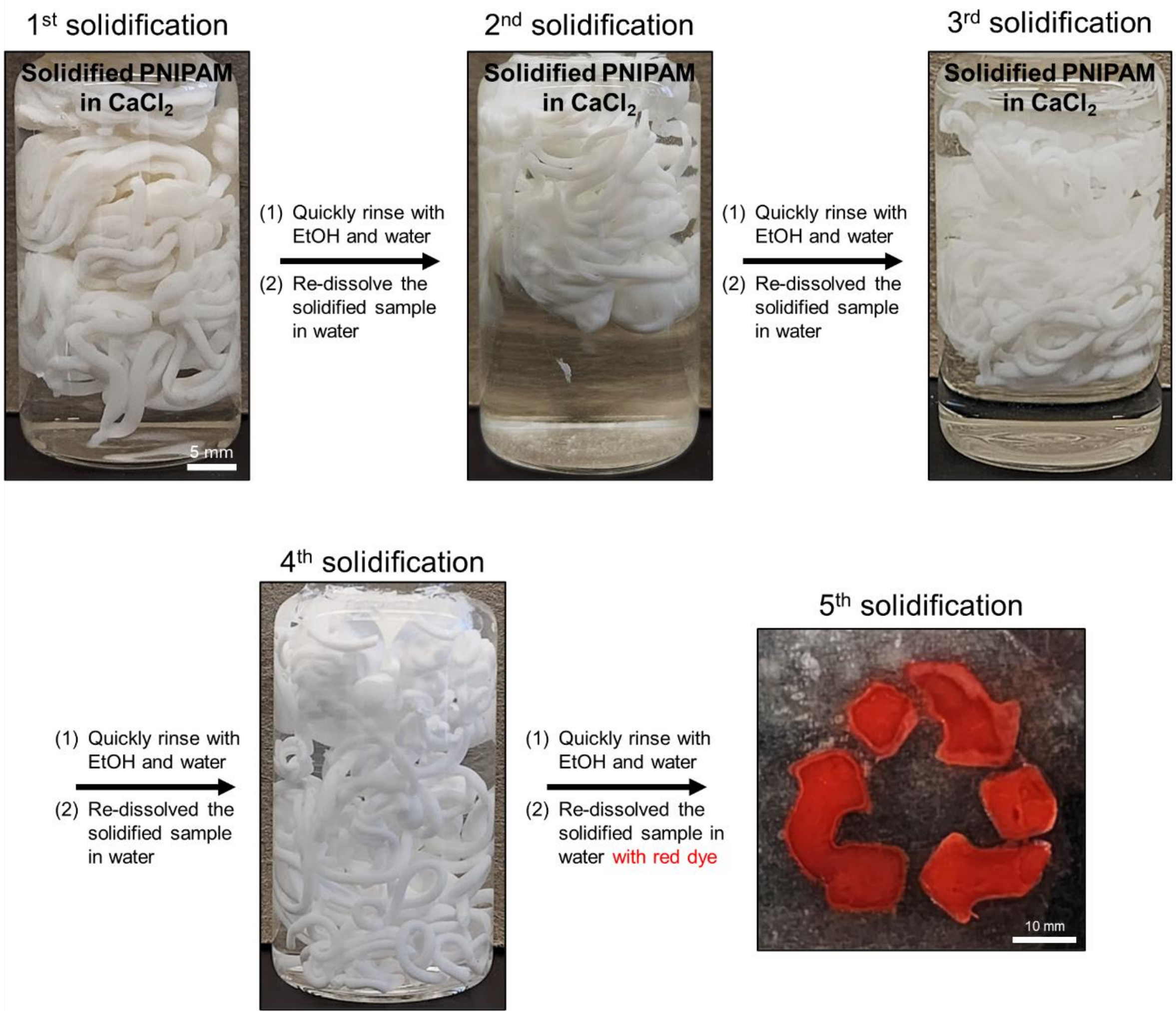

Supplementary Fig. 12. Recyclability of PNIPAM ink in multiple solidification–dissolution cycles. The solidified PNIPAM in $CaCl_2$ was quickly rinsed with EtOH and then water to remove excessive salt ions and subsequently re-dissolved in water at a 5 °C refrigerator. The dissolved PNIPAM was loaded into a syringe and extruded into $CaCl_2$ for the next solidification. Such a series of procedures was repeatable. In the 5th cycle demonstration, we added red dye for better visualization and then printed the shape of a recycle sign.

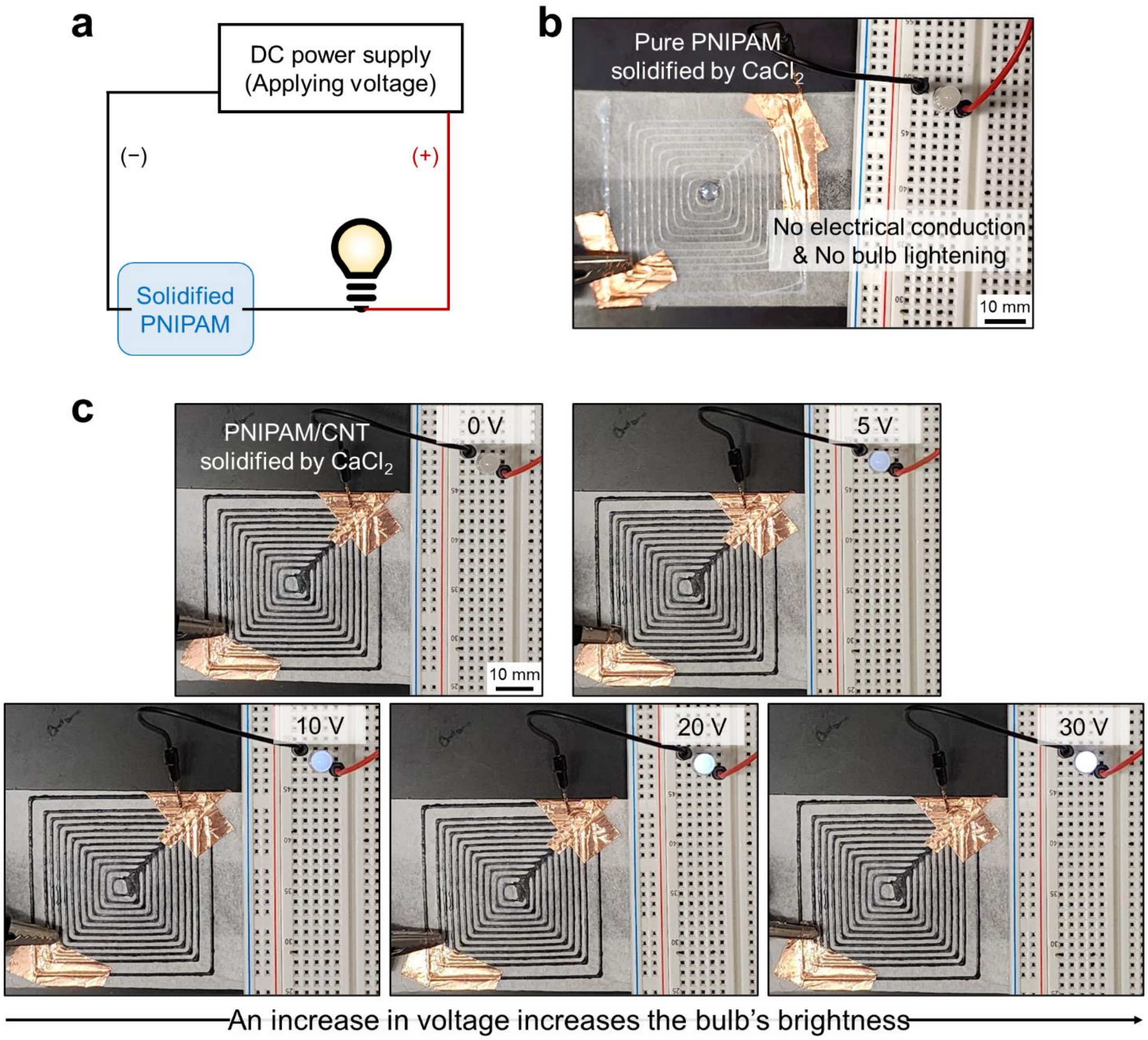

Supplementary Fig. 13. Conductivity comparison between pure PNIPAM and PNIPAM/ CNT composite. (a) Experimental setup. The printed and solidified PNIPAM structures (by 3 M $CaCl_2$), completely dried to remove the effect of ion conduction, were connected to the power supply that applied voltage across the entire circuit. The PNIPAM structures were connected to a bulb linked to the power supply. Copper wires were tightly attached to the PNIPAM structures with copper tape. (b) While the pure PNIPAM structure did not make a light bulb work (even at 30 V) due to insufficient conductivity, (c) the PNIPAM/CNT composite structure was able to power a light bulb. The brightness gradually increased with an increase in the applied voltage.

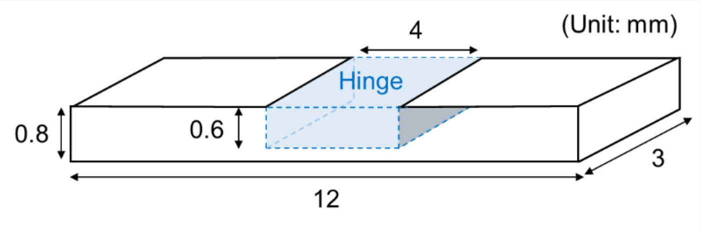

Supplementary Fig. 14. Schematic illustration of folding actuator structure. The bottom matrix was PNIPAM/nanoclay composite hydrogel and the hinge was L-PNIPAM/alginate composite solution.

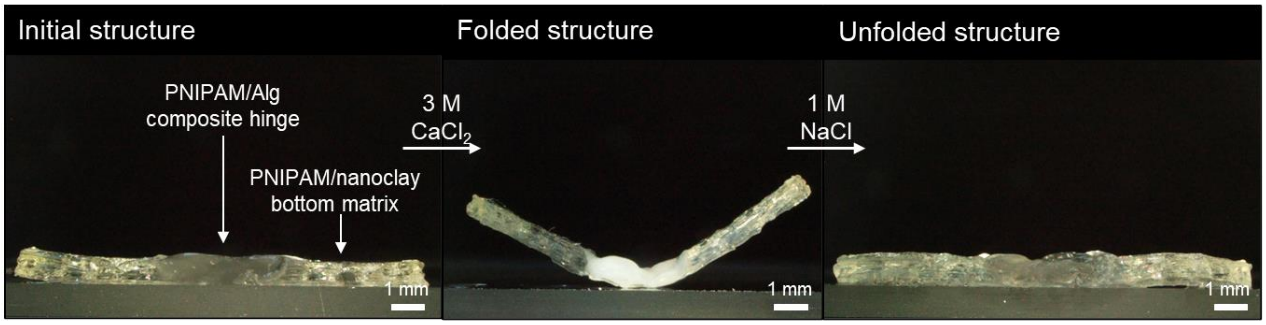

Supplementary Fig. 15. Self-folding and unfolding actuator comprising a hinge of PNIPAM/Alg composite solution. The volume contraction caused by crosslinking of PNIPAM/Alg hinge material at the 3 M $CaCl_2$ condition resulted in the folding. Next, the de-crosslinking of the hinge material at 1 M NaCl condition released the volume contraction of the hinge, thereby unfolding the actuator in a single stage.

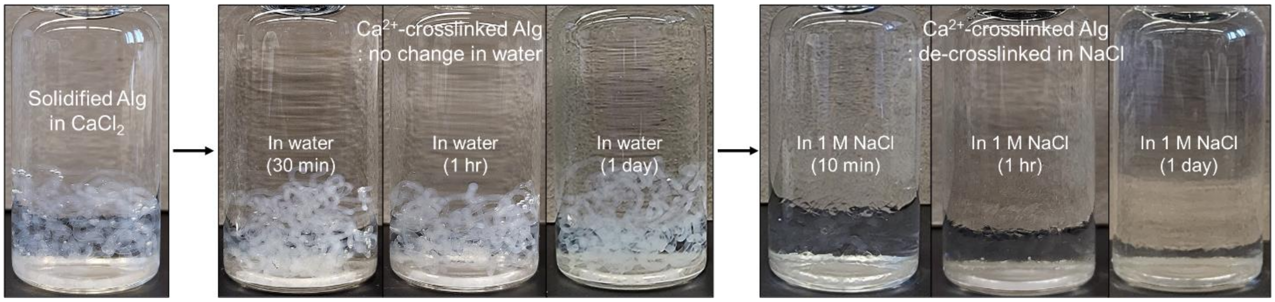

Supplementary Fig. 16. De-crosslinking of solidified Alg with Ca^{2+} crosslinking. The Ca^{2+}-crosslinked Alg was not de-crosslinked and dissolved in water but was able to be de-crosslinked and dissolved in 1 M NaCl solution.

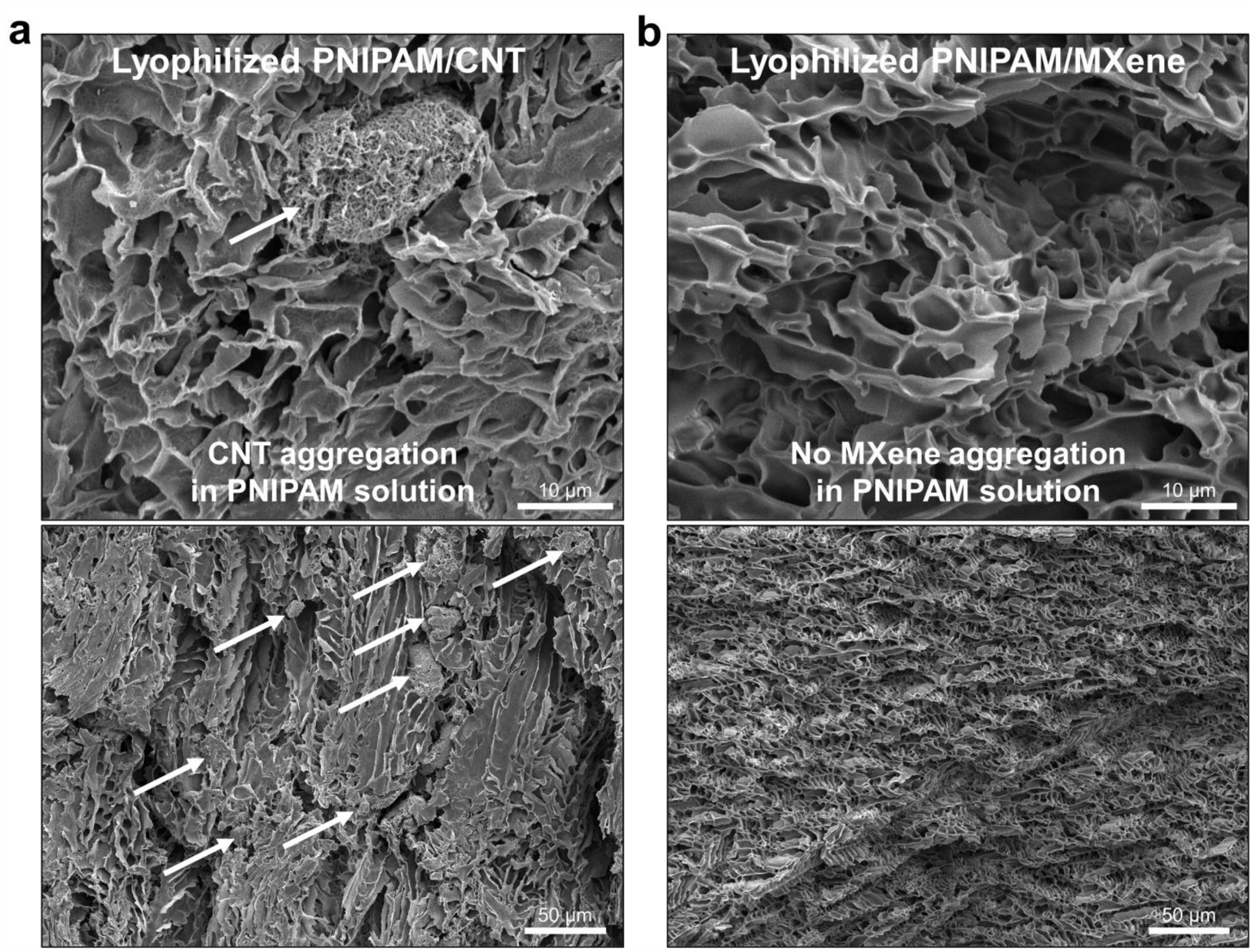

Supplementary Fig. 17. Cross-sectional SEM images of lyophilized PNIPAM/CNT and PNIPAM/MXene solutions. (a) Hydrophobic CNTs with long lengths (5–9 μm according to the manufacturer) were aggregated as denoted as arrows. (b) Hydrophilic MXene was homogenously dispersed into the solution. As observed in these cross-sectional SEM images (in lyophilized samples), the internal structure of the PNIPAM/MXene was similar to that of the pure PNIPAM displayed in Figure 1g.
