# Supplementary material for: Sustainable 3D printing by reversible salting-out effects with aqueous salt solutions
Source: Nat Commun. 2024 May 9;15:3925. doi: 10.1038/s41467-024-48121-7 (PMC11082145; doi:10.1038/s41467-024-48121-7)
Supplement: Supplementary file 3 — Description of Additional Supplementary Files [file 41467_2024_48121_MOESM3_ESM.pdf]

Description of Additional Supplementary Files

Supplementary Movie Legend:

Supplementary Movie 1. Embedded 3D printing of PNIPAM/CNT solution ink in the support 25 bath comprising Pluronic F-127 and CaCl2.
